# Association of four insulin resistance genes with type 2 diabetes mellitus and hypertension in the Chinese Han population

**DOI:** 10.1007/s11033-013-2937-0

**Published:** 2014-01-14

**Authors:** Bo Jiang, Ya Liu, Yuxin Liu, Fang Fang, Xue Wang, Bo Li

**Affiliations:** 1 Hospital Management Office & School of Public Health, Jilin University, Changchun, China; 2 Graduate School, Jilin University, Changchun, China; 3 School of Public Health, Jilin University, Changchun, China

**Keywords:** Type 2 diabetes mellitus, Hypertension, Polymorphisms, *ADIPOQ*, *LEPR*

## Abstract

Insulin resistance plays an important role in the development of type 2 diabetes mellitus (T2DM) and hypertension. The purpose of the present study was to evaluate the association between four insulin resistance genes (*ADIPOQ*, *LEPR*, *RETN*, and *TRIB3*) and both T2DM and hypertension. A total of 768 Han Chinese subjects were recruited into this study, including 188 cases who had T2DM alone, 223 cases who had hypertension alone, 181 cases with both T2DM and hypertension, and 176 control subjects with neither T2DM nor hypertension. Twenty-three tag SNPs in four insulin resistance genes were genotyped and analyzed for association with T2DM and hypertension. One intron SNP (rs13306519) in *LEPR* and one 3′UTR SNP (rs1063537) in *ADIPOQ* demonstrated a significant association with T2DM (*P* = 0.024 and 0.014 respectively). Another intron SNP (rs12037879) in *LEPR* and a promoter region SNP (rs266729) in *ADIPOQ* were significantly associated with hypertension (*P* = 0.041 and 0.042, respectively). These associations survived the permutation test (*P* = 0.023, 0.018, 0.026, and 0.035, respectively). These associations were still found to be significant in the additive model after adjusting for potential confounding factors including age, sex, BMI, HDL, LDL, total cholesterol, and triglyceride levels (*P* = 0.024, 0.016, 0.04, and 0.043, respectively). No other gene variants were found to be significantly associated with T2DM or hypertension (*P* > 0.05). None of the studied gene variants were found to be significantly associated with T2DM+ hypertension (*P* > 0.05). A significant interaction was observed between two SNPs rs13306519 in *LEPR* and rs266729 in *ADIPOQ* for T2DM (*P_int* = 0.012, OR_int = 2.67) and hypertension (*P_int* = 0.0041, OR_int = 2.23). These findings suggest that variants in *ADIPOQ* and *LEPR* are risk factors for T2DM and hypertension in the Chinese population and that variants in *RETN* and *TRIB3* are not major risk factors for these diseases.

## Introduction

Both diabetes and hypertension are important public health issues throughout the world. To date, at least 285 million individuals are affected by diabetes (with type II diabetes, type 2 diabetes mellitus (T2DM), accounting for 80–90 % of all cases), and that number is expected to reach 438 million by the year 2030 [1]. From 1994 to 2007, the prevalence of diabetes increased from 2.5 to 9.7 % in China. This has been attributed the aging of the population, urbanization, and lifestyle changes [2]. Hypertension affects nearly 27 % of the population globally and is predicted to increase by about 60 % to a total of 1.56 billion people in 2025 [3]. Hypertension affects 18 % of Chinese adults [4]. High blood pressure is reported in over two-thirds of patients with T2DM, and its development coincides with the development of hyperglycemia [5].

Genetic and environmental factors and their interactions play an important role in the pathogenesis of T2DM and hypertension [6]. It is assumed that glucose tolerance and blood pressure are under the control of a large number of genes, each of which has a relatively mild effect alone. To date, ~40 genetic loci for T2DM and 50 genetic loci for hypertension have been identified in genome-wide association studies [7–9]. Because all these known loci account for only 10 % of the susceptibility to T2DM and hypertension, candidate gene studies remain a valid and efficient approach to the identification of new genes and confirmation of the associations between known genes and both T2DM and hypertension in different populations. The use of association studies with SNP tags throughout candidate genes based on their physiological functions is expected to be a useful strategy for the identification of genes that contribute to this complex condition.

Reduced glucose tolerance and high blood pressure are closely associated. Both contribute to cardiovascular morbidity and mortality. Many pathophysiological mechanisms have been proposed for this association. Of these, insulin resistance is considered the most plausible and has drawn a great deal of attention. Genes encoding proteins that can modulate insulin signaling or action are, by definition, excellent candidates for the risk modulation of T2DM and hypertension.

Adipose tissue is considered an endocrine organ. It can regulate whole-body metabolism and both inflammatory and immune responses [10]. These actions are mediated by numerous adipose-tissue-derived hormones, collectively known as adipokines. These include adiponectin, leptin, and resistin. The discovery of these endocrine functions of adipose tissue has prompted the hypothesis that a genetically influenced dysregulation of the adipokine network may contribute to the pathogenesis of insulin resistance and related disorders such as T2DM and hypertension. Adiponectin is a 30 kDa adipocyte-secreted hormone. It is involved in regulation of blood glucose levels, insulin sensitivity, and lipid metabolism [11, 12]. The *ADIPOQ* gene, which is located on chromosome 3q27, encodes adiponectin. It has been identified as a susceptibility locus for T2DM in genome-wide linkage studies and genome-wide association studies [13–16]. The *ADIPOQ* gene has also been implicated in the etiology of hypertension [17]. The leptin receptor (LEPR) is a single-transmembrane-domain receptor of the cytokine-receptor family. It is distributed widely in the tissue and it has several alternatively spliced isoforms [18]. There is growing evidence indicating that LEPR may play a wide role in human metabolism of insulin, glucose, and triglycerides. The participation of leptin in the hypothalamus has been reported to increase blood pressure through the LEPR [19]. Recent studies have identified associations between variants in the *LEPR* gene and both T2DM and blood pressure [20–24]. Resistin is a macrophage-derived signaling polypeptide hormone. In humans it has a molecular weight of 12.5 kDa and a length of 108 amino acids. Previous animal studies, have suggested that resistin may play an important role in the pathogenesis of insulin resistance [25, 26]. The *resistin* (*RETN*) gene, located on 19p12.2, has been associated with T2DM in several prospective epidemiological studies across a wide variety of population groups, but results have been conflicting [27] Studies on biological function, epidemiology, and genetics, indicate that *ADIPOQ*, *LEPR*, and *RETN* may be associated with T2DM and hypertension.

Tribbles homolog 3 (TRIB3) is a 45 kDa pseudokinase, involved in the impairment of insulin signaling by affecting insulin-induced Akt activation in several insulin target tissues [28]. The *TRIB3* gene, located on chromosome 20p13, is associated with T2DM [29, 30]. This makes *TRIB3* an excellent candidate for T2DM. In addition, because insulin resistance is the physiopathologic foundation of more than one metabolic syndrome, *TRIB3* gene may also be associated with high blood pressure.

In the present study, we evaluated the association between 4 insulin resistance genes (*ADIPOQ*, *LEPR*, *RETN*, and *TRIB3*) and both T2DM and hypertension. We found several SNPs in *LEPR* and *ADIPOQ* to be significantly associated with T2DM and hypertension.

## Methods

### Cases and control subjects

The study protocol was approved by the Ethics Committee for Human Research of Jilin University. Informed consent was obtained from all participants after explanation of the nature and possible consequences of the study. In the survey, verbal consents were recorded but no written consent. Because the persons are old and most illiterate, well-trained clerks presented at the discussion with the patient and made independent record of his or her observations with impartial witness. Jilin University Ethics Committee approved this procedure.

A total of 768 Chinese subjects were recruited into this study, including 188 cases with T2DM alone, 223 cases with hypertension alone, 181 cases with both T2DM and hypertension, and 176 control subjects with neither T2DM nor hypertension (Table 1). The diagnosis of T2DM was based on clinical and laboratory criteria as defined by the World Health Organization in 1999. We excluded patients with MODY and type I diabetes using fasting blood glucose, clinical information, and personal patient histories. None of the patients with T2DM had ever had ketoacidosis. The T2DM treatment included oral anti-diabetic drugs and insulin. Blood pressure was measured after a 10 min rest at a sitting position. Hypertension was defined as a mean systolic blood pressure >140 mmHg and/or a mean diastolic blood pressure >90 mmHg. Patients taking any antihypertensive medication were defined as hypertensive. Exclusion criteria included the presence of any of secondary cause of hypertension, such as chronic renal disease, renal arterial stenosis, primary aldosteronism, contraction of the aorta, thyroid disorders, Cushing syndrome, and pheochromocytoma. The presence of these criteria were confirmed through extensive clinical examinations and investigations (including blood chemistry, renal function tests, endocrine examination, and abdominal sonogram). Control subjects were collected from the same geographic regions as the patients with T2DM and hypertension and had similar ethnic backgrounds. They had no known personal or family history of diabetes or hypertension. Demographic data including age, sex, weight, height, duration of diabetes, and hypertension or current use of anti-hypertensive medications were recorded via interviewer-administered questionnaires. All subjects were Han Chinese from northeastern China.

**Table 1:** Demographic and clinical features of the study subjects

Group	DM	HT	DM + HT	Control subjects
Number of subjects	188	223	179	176
Gender (M/F)	62/126	67/156*	59/120	75/101
Age at examination (years)	67.9 ± 6.7	68.8 ± 6.7*	68.2 ± 6.1	67.1 ± 7.1
Body mass index (kg/m^2^)	24.1 ± 4.0*	24.5 ± 4.3*	26.0 ± 3.7*	23.1 ± 3.3
Systolic blood pressure (mmHg)	128.1 ± 17.3*	154.6 ± 21.0*	153.1 ± 19.9*	121.3 ± 10.9
Diastolic blood pressure (mmHg)	78.6 ± 9.8	90.2 ± 10.1*	88.5 ± 12.4*	76.8 ± 7.8
Glucose (mmol/l)	7.4 ± 2.0*	5.3 ± 0.5	7.1 ± 1.7*	5.3 ± 0.5
HDL (mmol/l)	1.4 ± 0.4	1.4 ± 0.4	1.5 ± 0.5*	1.4 ± 0.4
LDL (mmol/l)	3.3 ± 0.9	3.3 ± 0.9	3.3 ± 0.9	3.2 ± 0.8
Total cholesterol (mmol/l)	5.2 ± 1.1	5.2 ± 1.2	5.3 ± 1.2	5.1 ± 0.9
Triglycerides (mmol/l)	1.9 ± 1.0*	1.7 ± 0.8	1.9 ± 0.9*	1.6 ± 0.99

*DM* represents type 2 diabetes, *HT* represents hypertension

* *P* < 0.05 versus controls

### Polymorphisms and genotyping

Venous blood samples were collected in EDTA-containing tubes from all participants after an overnight fast of at least 10 h. Genomic DNA was extracted from peripheral blood leukocytes. Tag SNPs were selected to cover the whole gene regions of *ADIPOQ*, *LEPR*, *RETN*, and *TRIB3* according to the HapMap Han Chinese population (Phase II + III, release 27; Table 2). The tag SNPs located in exons and promoter regions were selected in higher priority. The selected tag SNPs captured all SNPs from 5 kb upstream to 5 kb downstream of the respective genes with r^2^ > 0.8 and minor allele frequency (MAF) >0.1. SNP genotyping was performed by Bio Miao Biological Technology Co. Ltd. (Beijing, China) using a MassARRAY system (Sequenom, San Diego, CA, USA) with the iPLEX assay.

**Table 2:** Characteristics and genotype counts of the 26 variants in 4 insulin resistance genes

Gene	SNP	Chr	Position (bp)^a^	Location	Alleles (A/B)^b^	Genotype Count (AA/AB/BB)^b^			
						DM	HT	DM + HT	Control subjects
*LEPR*	rs12037879	1	65,942,707	Intron 1	A/G	8/55/106	8/68/108	6/57/97	2/45/104
*LEPR*	rs7554485	1	65,945,906	Intron 1	C/T	1/21/118	4/22/111	0/23/105	1/22/88
*LEPR*	rs1137100	1	66,036,441	Exon 2	A/G	1/40/96	2/35/98	2/41/88	3/35/72
*LEPR*	rs13306519	1	66,037,929	Intron 3	G/C	7/58/110	6/62/126	7/50/110	4/35/117
*LEPR*	rs1137101	1	66,058,513	Exon 4	A/G	3/34/140	5/42/146	1/31/133	3/33/117
*LEPR*	rs8179183	1	66,075,952	Exon 9	C/G	0/15/117	1/14/114	0/19/109	0/9/96
*ADIPOQ*	rs16861194	3	186,559,425	5′NEAR	G/A	9/43/127	5/55/138	4/47/118	7/46/109
*ADIPOQ*	rs266729	3	186,559,474	5′NEAR	G/C	11/69/96	23/90/85	12/68/93	11/64/84
*ADIPOQ*	rs12495941	3	186,568,180	Intron 1	T/G	35/78/63	24/101/74	25/88/56	20/80/57
*ADIPOQ*	rs2241766	3	186,570,892	Exon 1	G/T	14/75/91	14/86/102	12/67/90	10/65/91
*ADIPOQ*	rs1501299	3	186,571,123	Intron 2	A/C	13/69/88	18/58/109	12/65/84	7/68/74
*ADIPOQ*	rs1063537	3	186,574,075	3′UTR	T/C	11/59/81	45/79/70	18/57/75	25/56/65
*ADIPOQ*	rs12629945	3	186,577,127	3′NEAR	A/G	9/49/98	9/56/99	5/42/98	6/46/81
*ADIPOQ*	rs6444175	3	186,579,744	3 NEAR	A/G	15/72/95	19/65/113	12/70/88	6/78/78
*RETN*	rs2161490	19	7,731,954	5 NEAR	T/C	28/85/68	36/102/64	34/83/55	31/78/56
*RETN*	rs3745368	19	7,735,297	3 UTR	A/G	6/43/131	4/52/144	3/44/125	4/35/122
*TRIB3*	rs12626158	20	370,150	Intron 2	A/G	9/61/108	23/56/127	12/59/97	9/48/98
*TRIB3*	rs6051637	20	371,972	Exon 2	T/C	7/66/103	4/60/130	6/50/112	7/46/105
*TRIB3*	rs6115830	20	377,226	Exon 3	T/C	16/77/87	23/85/94	14/72/85	20/72/70
*TRIB3*	rs2295491	20	377,979	3′UTR	A/G	0/37/135	5/47/137	0/43/122	5/36/112
*TRIB3*	rs2295492	20	378,242	3 NEAR	C/G	6/46/120	7/54/130	5/39/121	4/49/100
*TRIB3*	rs6037542	20	382,876	3 NEAR	T/G	11/78/86	19/93/77	17/73/77	13/76/65

*DM* represents type 2 diabetes, *HT* represents hypertension

^a^Chromosomal position is based on NCBI Build 37.3 (National Center for Biotechnology Information, Bethesda, MD, USA)

^b^A represents the minor allele and B represents the common allele

### Statistical analysis

Demographic and clinical characteristics were compared between cases and control subjects by using either the chi square test for categorical data or unpaired *t* test for numerical data as appropriate. Data analyses for SNPs were performed using PLINK (v1.07) [31]. Hardy–Weinberg equilibrium was assessed by using the chi square test. Minor allele frequencies of each SNP between cases and control subjects were compared using Fisher’s exact test. The odds ratio (OR) and 95 % confidence interval (CI) were calculated using logistic regression. A permutation test was performed using the label-swapping and max(T) procedures for 10,000 permutations. The associations between the SNPs and the diseases were further evaluated using logistic regression after adjusting for potential confounding factors including age, sex, BMI, HDL, LDL, total cholesterol, and triglyceride levels. An additive effects model was applied to analysis of allele dosage in which the genotypes AA, AB, BB were coded as 0, 1, and 2, respectively. A represents the rare allele and B represents the common allele. Pairwise SNP–SNP interactions were analyzed using logistic regression. Multiple comparisons were corrected using the Bonferroni method.

## Results

Demographic and clinical characteristics of the study subjects are summarized in Table 1. There was a significant difference in gender (*P* = 0.01) and age (*P* = 0.02) between cases with hypertension and control subjects.

All SNPs were found to be in Hardy–Weinberg equilibrium in the control group (*P* > 0.01). One intron SNP (rs13306519) in *LEPR* and one 3′UTR SNP (rs1063537) in *ADIPOQ* demonstrated a significant association with T2DM (*P* = 0.024 and 0.014 respectively; Table 3). The allele G frequency of rs13306519 was higher in cases with T2DM than in the control subjects (20.6 vs. 13.8 %; OR = 1.62; 95 % CI, 1.07–2.45). The allele T frequency of rs1063537 was lower in cases with T2DM than in the control subjects (26.8 vs. 36.3 %; OR = 0.64; 95 % CI, 0.45–0.91). Another intron SNP (rs12037879) in *LEPR* and a promoter region SNP (rs266729) in *ADIPOQ* were significantly associated with hypertension (*P* = 0.041 and 0.042 respectively; Table 3). The allele A frequency of rs12037879 was higher in cases with hypertension than in the control subjects (22.8 vs. 16.2 %; OR = 1.53; 95 % CI, 1.03–2.26). The allele G frequency of rs266729 was higher in cases with hypertension than in the control subjects (34.3 vs. 27.0 %; OR = 1.41; 95 % CI, 1.02–1.95). These associations survived the permutation test (*P* = 0.023, 0.018, 0.026, and 0.035, respectively). These associations remained significant in the additive model after adjusting for potential confounding factors including age, sex, BMI, HDL, LDL, total cholesterol, and triglyceride levels (*P* = 0.024, 0.016, 0.04, and 0.043, respectively). No other gene variants were found to be significantly associated with T2DM and hypertension (*P* > 0.05). None of the gene variants studied here was found to be significantly associated with T2DM+ hypertension (*P* > 0.05).

**Table 3:** Single-SNP association of the studied genes with T2DM, hypertension, and both

Gene	SNP	Minor allele	MAF				P*		
			DM	HT	DM + HT	Control subjects	DM	HT	DM + HT
*LEPR*	**rs12037879**	A	0.210	0.228	0.216	0.162	0.13	**0.041**	0.10
*LEPR*	rs7554485	C	0.082	0.110	0.090	0.108	0.36	1.00	0.54
*LEPR*	rs1137100	A	0.153	0.144	0.172	0.186	0.34	0.22	0.72
*LEPR*	**rs13306519**	G	0.206	0.191	0.192	0.138	**0.024**	0.07	0.07
*LEPR*	rs1137101	A	0.133	0.135	0.100	0.128	0.63	0.82	0.32
*LEPR*	rs8179183	C	0.057	0.062	0.074	0.043	0.53	0.41	0.17
*ADIPOQ*	rs16861194	G	0.170	0.164	0.163	0.185	0.62	0.49	0.47
*ADIPOQ*	**rs266729**	G	0.259	0.343	0.266	0.27	0.73	**0.042**	0.93
*ADIPOQ*	rs12495941	T	0.421	0.374	0.408	0.382	0.34	0.88	0.52
*ADIPOQ*	rs2241766	G	0.286	0.282	0.269	0.256	0.39	0.45	0.73
*ADIPOQ*	rs1501299	A	0.279	0.254	0.276	0.275	0.93	0.54	1.00
*ADIPOQ*	**rs1063537**	T	0.268	0.436	0.310	0.363	**0.014**	0.06	0.19
*ADIPOQ*	rs12629945	A	0.215	0.226	0.179	0.218	1.00	0.84	0.29
*ADIPOQ*	rs6444175	A	0.280	0.261	0.277	0.278	1.00	0.67	1.00
*RETN*	rs2161490	T	0.390	0.431	0.439	0.424	0.35	0.88	0.76
*RETN*	rs3745368	A	0.153	0.150	0.145	0.134	0.51	0.59	0.74
*TRIB3*	rs12626158	A	0.222	0.248	0.247	0.213	0.85	0.29	0.35
*TRIB3*	rs6051637	T	0.227	0.175	0.185	0.19	0.25	0.62	0.92
*TRIB3*	rs6115830	T	0.303	0.324	0.292	0.346	0.25	0.58	0.16
*TRIB3*	rs2295491	A	0.108	0.151	0.130	0.15	0.13	1.00	0.49
*TRIB3*	rs2295492	C	0.169	0.178	0.149	0.186	0.61	0.84	0.20
*TRIB3*	rs6037542	T	0.286	0.347	0.320	0.331	0.24	0.69	0.80

Significant SNPs and *P* values are shown in bold type

*DM* represents type 2 diabetes, *HT* represents hypertension

* Obtained from Fisher’s exact test versus control subjects

SNP–SNP interaction analysis identified a significant interaction between two SNPs rs13306519 in *LEPR* and rs266729 in *ADIPOQ* for T2DM (*P_int* = 0.012, OR_int = 2.67) and hypertension (*P_int* = 0.0041, OR_int = 2.23).

## Discussion

In present study, we evaluated 23 tag SNPs in 4 critical insulin resistance genes for association with T2DM and hypertension in the Chinese Han population. We found one SNP in *ADIPOQ*, rs1063537, and one SNP in *LEPR*, rs13306519, to be significantly associated with T2DM. An SNP in *ADIPOQ*, rs266729, and another SNP in *LEPR*, rs12037879, were significantly associated with hypertension. These findings suggest that *ADIPOQ* and *LEPR*, which encode proteins associated with insulin resistance, may play an important role in the development of T2DM and hypertension.

*ADIPOQ* is considered a gene for T2DM and metabolic syndrome. The concentration of adiponectin in the plasma has been suggested to play an important role in the modulation of insulin sensitivity and glucose homeostasis. It has also been found to be decreased in patients with T2DM, hypertension, dyslipidemia, coronary artery disease, and obesity [32–37]. All of these conditions are closely related to insulin resistance. In line with plasma levels of adiponection, the *ADIPOQ* gene has been identified as a susceptibility locus for the metabolic syndrome, T2DM, and cardiovascular disease. However, studies of the associations between the *ADIPOQ* gene with adiponectin level and the metabolic syndrome often show conflicting results [38, 39]. Studies on the associations between *ADIPOQ* SNPs with T2DM and hypertension in Chinese populations have also produced conflicting results [17, 40–42]. SNP rs2241766 in exon 2 and SNP rs1501299 in intron 2 are the most widely investigated variants in *ADIPOQ*. At least 19 studies have shown SNP rs2241766 to be associated with T2DM in the Chinese population, but no association was found in any of 8 other studies [40]. A recent large-scale meta-analysis of 39 studies showed that SNPs rs2241766 and rs1501299 are not associated with T2DM in either Asian or European populations [42]. A meta-analysis of 24 studies suggested no significant association between SNPs rs2241766 and rs1501299 and hypertension in Chinese individuals [41]. A large cardiovascular risk factor prevalence study also failed to find any association between SNPs rs2241766 and rs1501299 and hypertension [17]. In the present study, we did not find any significant association between these 2 SNPs and either T2DM or hypertension, which is consistent with previous studies [41, 42]. Intriguingly, three polymorphisms (rs16861194, rs17300539, rs266729) in *ADIPOQ* were associated with risk of T2DM in European populations but none of these polymorphisms were associated with risk of T2DM in Asian populations [42]. In the present study, no significant association was observed between SNPs rs16861194 and rs266729 with T2DM in a Chinese Han population. Taken together, these findings suggest that ethnically specific polymorphisms might contribute to the development of T2DM and hypertension in different populations.

In the present study, we found the G allele of a promoter SNP rs266729 in *ADIPOQ* to be significantly associated with the increased risk of hypertension (OR = 1.41). A previous study in a Hong Kong Chinese population also suggested that genetic variants in the promoter region of *ADIPOQ* are associated with increased risk of hypertension [17]. The G allele of rs266729 has been linked to decreased serum levels of adiponectin [43]. Because adiponectin has been reported to exert an antiatherosclerotic effect, we might speculate that G allele of rs266729 can influence the abundance expressor function of adiponectin. Low levels of this peptide in serum can directly influence endothelial function. This hypothesis is supported by the observation that both the expression and activity of eNOS are increased in cultured vascular endothelial cells that have been treated with globular adiponectin [44]. This promoter polymorphism is also associated with T2DM in Chinese, French, German, and Swedish populations [22, 40, 45, 46]. One meta-analysis confirmed the association between rs266729 and T2DM, but another meta-analysis did not [39, 47]. The discrepancy between these results may be partially due to ethnic differences in the populations, selection criteria of the study subjects, and sample size.

It has been proposed that SNPs in the 3′UTR of *ADIPOQ*, like promoter SNPs, may remodel adiponectin conformation, causing T2DM [48]. In the present study, one 3′UTR SNP, rs1063537, found in *ADIPOQ*, was found to be significantly associated with T2DM. To the best of our knowledge, this is the first time that this SNP has been shown to be associated with T2DM. One previous study reported the T allele of rs1063537 to be associated with higher adiponectin levels in Caucasian women [49]. Other research groups found another 2 SNPs (rs1063539 and rs6773957) in 3′UTR of *ADIPOQ* to be associated with T2DM in the Chinese population [50, 51]. SNP rs6773957 was found to be associated with adiponectin level in a genome-wide association study [52]. The HapMap data in Asian samples show that rs1063537 is in linkage disequilibrium with rs1063539 and rs6773957.

Genetic variants in the *LEPR* gene have been reported to have a profound impact on body weight, insulin resistance, blood pressure, and other metabolic disease parameters [23, 38]. In the present study, we found one intron SNP, rs13306519, in *LEPR* to be significantly associated with T2DM and another intron SNP rs12037879 was found to be significantly associated with hypertension. To our knowledge, this is the first time that these two *LEPR* SNPs have been associated with T2DM and hypertension. Further studies in different populations are needed to confirm our findings.

The nonsynonymous SNP rs1137101 (Gln223Arg), which is in the *LEPR* gene, has been extensively investigated in a wide range of populations. The G allele of rs1137101 has been associated with lower blood pressure [23]. However, the A allele has been associated with increased insulin and leptin levels and the risk of developing T2DM [53]. Another two nonsynonymous SNPs, rs8179183 (Lys656Asn) and rs1137100 (Arg109Lys), have been reported to be associated with hypertension and T2DM [54, 55]. However, the results of published studies have been inconsistent [23, 53]. One study performed in the Chinese population showed rs1137101 to be associated with hypertension but not with T2DM [56]. In the present study, we did not find any association between these 3 SNPs with T2DM or hypertension. This could be partly explained by a lack of statistical power sufficient to detect variants that are likely to confer only a modest effect on T2DM and hypertension. Further studies with larger sample size and greater statistical power are required to confirm these associations.

Intriguingly, a significant interaction was identified between two SNPs rs13306519 in *LEPR* and rs266729 in *ADIPOQ* for both T2DM (*P_int* = 0.012, OR_int = 2.67) and hypertension (*P_int* = 0.0041, OR_int = 2.23), suggesting that individuals carrying variants of both genes might have a higher risk of developing T2DM and hypertension than those carrying variants in single genes.

The *RETN* and *TRIB3* genes have been found to be associated with T2DM in previous studies, but the results are often conflicting [27, 29, 30]. In the present study, no significant association was found between the *RETN* and *TRIB3* genes and any medical condition, suggesting that genetic variants in these genes may not be major risk factors for the development of T2DM or hypertension in the Chinese population.

It is crucial to avoid population stratification in genetic association studies. One of the strengths of the present study is the ethnically matched cases and control subjects. The observed association in our study is unlikely to have been affected by population stratification. Another major strength of our study is the relatively large sample size in both the disease and control groups. Although the sample size examined in the present work is smaller than those of other large-scale studies, the power calculations suggest that these samples have sufficient power to detect common variants with moderate genetic effects. Specifically, these samples for T2DM had 86.7 % power to detect variants with minor allele frequencies of 0.15 and OR of 1.6 at significance level of 0.05. The samples for hypertension had 91.7 % power to detect variants with minor allele frequencies of 0.15 and ORs of 1.6 at a significance level of 0.05. All four genes investigated in this study have been found to be associated with T2DM and hypertension in previous studies, but the results are often conflicting. The purpose of the present study was to evaluate these associations in a different population. Because this study was not a discovery study, we did not attempt to replicate our results in independent samples. One limitation of the present study was that we did not investigate rare variants but only SNP tags with a MAF >0.1. We therefore could not rule out the possibility of the existence of rare variants in these genes that might be associated with T2DM and hypertension. Our sample size would not provide sufficient power for a detection of the associations of these rare variants with T2DM and hypertension. A larger sample and resequencing of the whole gene coding regions would be required to elucidate the role of these rare variants in the development of T2DM and hypertension. Although the selected tag SNPs evaluated in this study are not sufficient to define fine structure of the haplotype block in this population, the purpose of the present study was to investigate the association of these genes with T2DM and hypertension. It was anticipated that these selected tag SNPs would be capable of capturing the majority of polymorphisms in these genes. As the associations we observed in the present study were nominally significant, it is possible that these associations are false positives. However, it is statistically impossible for a candidate gene study like this one to yield a very small *P* values like those reported in a genome-wide association studyies with thousands of samples. Permutation tests suggest that these nominal associations are likely to be true associations.

Serum levels of adipokines, including adiponectin, leptin, and resistin, have been reported to be involved in the development and progression of both T2DM and hypertension. Genetic variants in genes encoding these adipokines have been found to be associated with serum levels of these adipokines. It would be interesting to investigate the association between the gene variants and the serum levels of these adipokines. However, because serum levels of these adipokines were not measured in the present study, it is impossible to perform such an analysis as part of this project. Further studies are warranted.

In summary, the present findings suggest that variants in *ADIPOQ* and *LEPR* are significant risk factors for T2DM and hypertension. However, variants in *RETN* and *TRIB3* are not major risk factors for the development of these complex disorders, at least in the Chinese population.
